# Sudden perceived absence of foetal movement – a unique presentation of a vertebral haemangioma in pregnancy

**DOI:** 10.1259/bjrcr.20200199

**Published:** 2021-10-20

**Authors:** Ankitha Devaraj, Jamaal Raoof, Osman Janjua, Kevin Tsang, Muhammad Zamir

**Affiliations:** 1 Watford General Hospital, West Hertfordshire NHS Trust, Vicarage Road, Watford, Hertfordshire, United Kingdom; 2 Lincoln County Hospital, Greetwell Road, Lincoln, Lincolnshire, UK; 3 University Hospital Monklands, Monkscourt Avenue, Airdrie, UK; 4 St Mary’s Hospital, Praed Street, London, UK

## Abstract

**Objective:**

Symptomatic vertebral haemangiomas that present during pregnancy are extremely uncommon with few cases reported in literature. Epidural haemangiomas are rarer still with few documented.

**Methods:**

In this report, we describe the case of a 22-year-old pregnant patient who presented with apparent loss of foetal movement at 38 weeks’ gestation. Clinical review demonstrated the foetus was well but neurological examination revealed lower limb paresthesia, paresis and evident uterine hypoesthesia. An MRI scan illustrated a haemangioma in the T1 vertebral body with an epidural component causing cord compression.

**Results:**

The management of spinal haemangiomas that present during pregnancy is a complex clinical scenario, which requires careful multidisciplinary consideration to determine if surgical intervention is appropriate. In this case, the patient had an emergency caesarean section followed by posterior decompression and laminectomy of the T1 vertebra with excellent post-operative recovery.

**Conclusion:**

Gestational increase in the size of vertebral haemangiomas is well documented. We discuss a rare case in which a young pregnant patient presents with an atypical symptom of a vertebral haemangioma (uterine hypoesthesia). This case highlights the importance of prompt imaging in these scenarios and a cohesive multidisciplinary approach in order to provide optimal treatment for the patient.

## Introduction

Vertebral haemangiomas are benign, slow growing cavernous or capillary tumours. They are common in the female population and are mainly located in the thoracic spine, representing 10–12% of spinal tumours. They are symptomatic in only 1% of cases^1–3^ and are usually incidental findings on routine MRI scans for other diseases. The haemangioma can become symptomatic depending on the location and the size. There is no increase in incidence of haemangiomas in pregnancy as compared to the general population. Spinal haemangiomas are more likely to be identified during pregnancy due to haemodynamic and endocrinal changes during the third trimester, which plays a key role in the expansion and enlargement of haemangiomas.

The main symptoms include mid or upper back pain. The pain usually subsides after delivery in most patients; however, some cases can be more severe, and patients can present with lower paraesthesia, paraparesis or paraplegia as a result of spinal cord compression due to epidural extension or vertebral fracture.^4^ Prompt diagnosis is essential in order to provide optimal therapy and prevent morbidity for both the mother and foetus.^4^ A delay in surgical treatment can have a significant impact on neurological outcome. We present the case of a pregnant patient with a compressive vertebral haemangioma.

## Case summary

A fit and healthy 22-year-old presented with a sudden absence of foetal movement at 38 weeks’ gestation. Additional history revealed a two-week history of worsening leg weakness as well as back pain. She denied trauma or faecal and urinary incontinence. Her medical history was unremarkable apart from minor thoracic scoliosis.

Neurological assessment revealed mild bilateral weakness in the hip, knee and ankle flexion. Hip and knee extension were also weakened although ankle extension was preserved bilaterally. Brisk knee and ankle reflexes were noted with positive bilateral Babinski reflex. A reduction in nociception was demonstrated at the T3-T4 level with preservation of proprioception. Cranial nerve and upper limb examinations were normal.

The patient also underwent obstetric review comprising clinical, sonographic and cardiotocographic examination which confirmed that the foetus was well with normal movement present.

Her blood results were unremarkable. The patient was unable to tolerate an MRI scan; however, a limited *T*_2_-weighted sequence of the thoracic spine demonstrated a hyperintense lesion involving the T1 vertebral body, with abnormal soft tissue in the ventral epidural space, craniocaudal extension and bilateral involvement of the T1 and T2 intervertebral foramina, causing marked cord compression.

**Figure 1.**
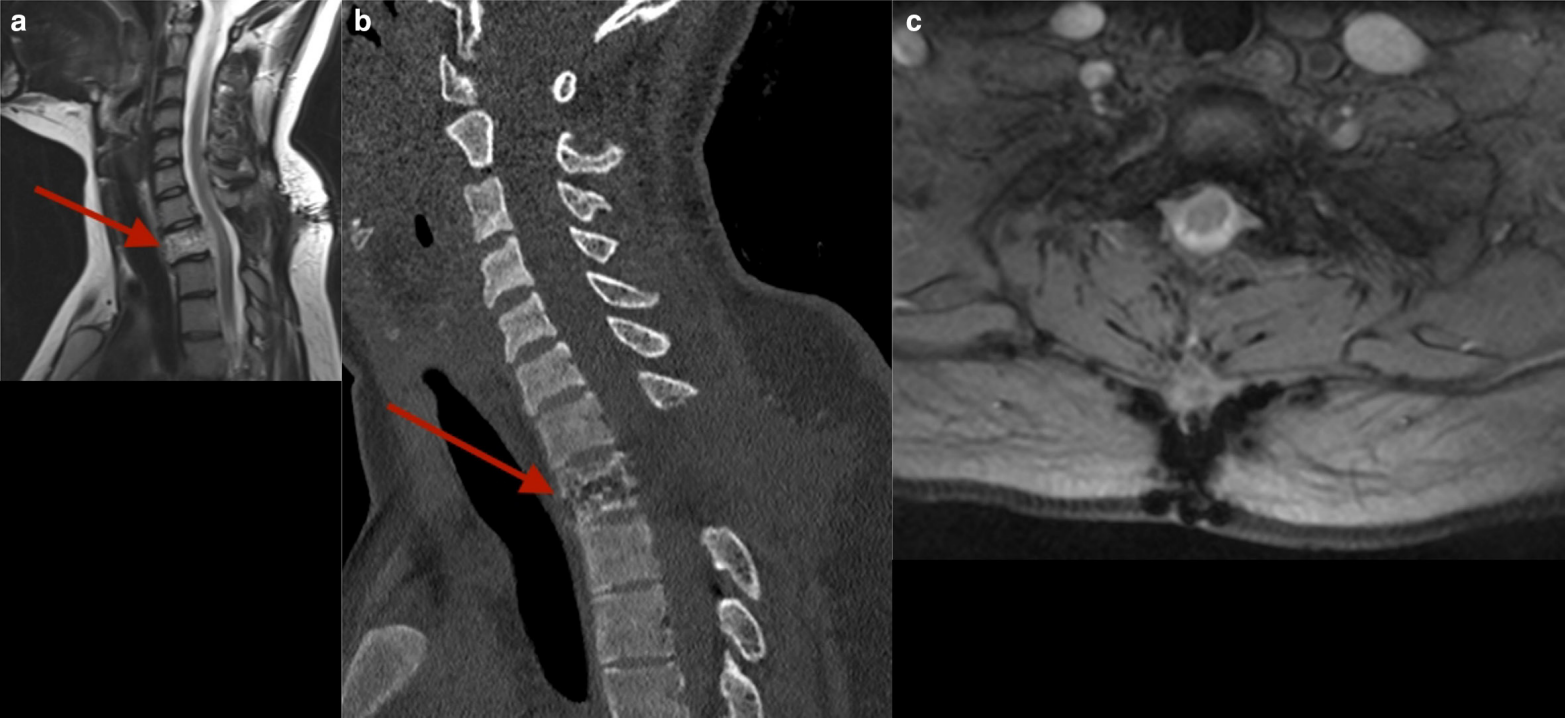
a. Postop sagittalT2 A sagittal slice from a *T*_2_-weighted sequence demonstrating the partially resected hyperintense haemangioma ( arrow) in the T1 vertebral body with resolution of cord compression following laminectomy b: Postop sagittal CT A sagittal image from a CT scan demonstrating the haemangioma and laminectomy at the level of the T1 vertebral body ( arrow). c: Postop Axial T2 gradient echo images An axial slice from a fat-saturated sequence highlighting the laminectomy at the level of the T1 vertebral body.

After a multidisciplinary team decision, the patient underwent an uncomplicated emergency caesarean section, followed by a posterior decompression with laminectomy of the T1 vertebral body (Figure 1a, b and c), along with the caudal half of C7 and the cranial half of T2. Multiple biopsies were taken from the epidural mass. Histopathological analysis confirmed a haemangioma with a soft tissue component but no malignant features. Pre-operative embolisation can reduce surgical blood loss; however in our case, there was no role for embolisation as this was a surgical emergency due to cord compression. The patient was clinically well following the procedure and discharged with a Miami J collar and thoracic brace with physiotherapy team follow-up.

## Discussion

*Guthkelch* described the first case of a symptomatic vertebral haemangioma associated with pregnancy in 1948. Since then, there have been more than 30 reported cases in literature, mostly during the third trimester, with only one case in the second trimester.^4^

Our case is unique primarily because of the unusual presenting symptom: the patient attended the obstetric department because of a perception of absent foetal movement. Sensory innervation of the uterus is via afferent fibres that travel to the inferior hypogastric plexus and enter the spinal cord as part of the T10 to L1 nerve roots. These subsequently ascend via the dorsal columns – related to deep touch, proprioception and vibration – and terminate in the thalamus. The patient suffered from compression of the spinal cord with a likely compromise of all the sensory and motor pathways, but it is not clear why uterine hypoesthesia was the primary complaint and there are no articles which have encountered or explored a similar scenario.

Haemangiomas consist of thin walled, blood-filled vessels and sinuses lined by endothelium, interspersed among the longitudinally (vertically) oriented trabeculae of bones.^5^ Accumulation of lipid material is a common secondary phenomenon.

Several hypotheses have been postulated for the presentation of vertebral haemangiomas in pregnant patients. Some authors hypothesise the haemangiomas may become more pervasive and increase in size during pregnancy due to a decrease in bone density, but this is not a widely held view. There are two main factors which are thought to lead to expansion of haemangiomas:

1. Haemodynamic: up to 50% increase in blood volume during pregnancy and venous pressure caused by compression of the inferior vena cava by the gravid uterus. This increases the intra-abdominal pressure which results in redistribution of blood flow particularly through the vertebral venous plexus.^1,4^ These features lead to an increase in volume of the haemangioma and associated mass effect.
2. Hormonal: increased progesterone and oestrogen levels during the third trimester cause endothelial proliferation and venous distensibility due to increase in plasma volume.^1,3,4^ Relaxin, a hormone produced by the placenta, possibly plays a role due to its vasodilatory effect.^1^

Vertebral haemangiomas can cause neurologic symptoms through a variety of mechanisms, including haemorrhage into the epidural space, expansion of an involved vertebrae with spinal canal narrowing, compression fracture of vertebrae, subperiosteal growth of tumour, and spinal cord ischaemia through a ‘steal-type’ phenomenon.^6^

MRI is the best modality to identify vertebral haemangiomas given its superior soft tissue resolution and also because it allows for fine evaluation of the cord. Additionally, the lack of ionising radiation is an important advantage given the radiosensitive nature of the foetus. A typical haemangioma demonstrates hyperintensity on T1 and *T*_2_-weighted sequences related to the presence of intralesional fat, blood vessels and interstitial oedema. Aggressive haemangiomas, so-called because of rapid expansion resulting in cord or neuronal compression, contain less fat and more vascular stroma compared to non-aggressive haemangiomas and therefore have decreased signal on T1 sequences. The extraosseous component may be hypointense relative to marrow on non-contrast *T*_1_-weighted sequences; uniform enhancement and T2 hyperintensity of osseous and extraosseous components are typical. Cortical erosion, extradural soft-tissue expansion of the posterior elements and invasion of the spinal canal are key radiological signs of aggression.^1^ Haemangiomas can demonstrate a range of appearances on spinal arteriography from normal vascularity to marked hypervascularity.^5^

Vertebral haemangiomas are typically characterised by a thickening of vertically striated trabeculae or an irregular honeycomb pattern on plain radiographs in contrast to other spinal tumours.^7^ Another distinguishing feature on CT is a ‘polka dot’ or ‘corduroy cloth’ pattern because of an overall decrease in marrow density due to the presence of fat.^5^

Morphological MR sequences might fail to differentiate between malignant and benign lesions because signal characteristics may overlap. Advanced MR sequences like dynamic contrast MR imaging and arterial spin labelling could be used to help with this differentiation. Diffusion-weighted MR imaging (DWI) and the derived apparent diffusion coefficient (ADC) maps may help to define the nature of the lesion, offering both qualitative and quantitative information about the haemangioma.^7^

Diffusion tensor imaging (DTI) is an emerging MR imaging technique for evaluating the microstructure of biologic tissues. *Razek et al* confirmed DTI can be used for the differentiation of malignant and benign vertebral collapse. This study showed an increase in the diffusivity of bone marrow in patients with benign bone marrow disease in comparison to patients with malignant pathological involvement.^7^

Indication for treatment includes severe back pain or neurological impairment. Radiation therapy and embolisation involving the use of ionising radiation are contraindicated during pregnancy due to the obvious risks to the foetus. Surgery such as vertebroplasty and intralesional ethanol injections are being used for the management of symptomatic haemangiomas.^1,8^ A series of 11 cases treated with ethanol ablation alone found total regression of the lesions on post-procedure angiography with no recurrence at follow-ups up to 76 months after treatment.^8^ In our case study, a laminectomy was undertaken which allowed for cord decompression and neurological recovery. Anterior corpectomy should be considered with caution in pregnancy since haemangiomas are highly vascular.

The optimal timing of surgery is controversial; however, important factors such as gestation, maturity of the foetus and seriousness of maternal neurologic deficits should be considered. The general recommendation is that surgical resection of symptomatic vertebral haemangiomas during pregnancy be delayed until a viable foetus can be delivered.^9^*Y.Kiroglu et al* noted many patients had spontaneous remission after delivery; however, symptoms did recur, requiring surgery at a later date. For patients at 36 weeks’ of gestation or later, spinal surgery after induction of delivery or a caesarean delivery can be performed safely.^9^

*Chi et al* recommended observation for asymptomatic patients at >32 weeks’ of gestation and surgery for patients with severe neurological deficits even at <32 weeks’ of gestation.^10–12^*Wang et al* recommended surgery for patients with severe or rapidly developing neurological deficits (power less than 3/5), especially for those with sphincter disturbance and vertebral fracture, irrespective of the trimester. Before surgery, the patients should first be evaluated by obstetricians for the possibility of vaginal delivery or caesarean section.^11^

Only a few haemangiomas with defined epidural components – whether originating or by extension – have presented with cord compression in pregnancy and this is a distinctive feature in our case. It is unusual for a haemangioma to be located purely in the epidural space, this being defined as the presence of 90% of the tumour volume in the epidural location.^13^ Most haemangiomas cause compression as a result of osseous expansion or epidural haemorrhage.

Furthermore, we believe this is the first case report of a compressive haemangioma in a pregnant patient presenting with uterine hypoesthesia and resulting in a perception that foetal motion was absent.

## Conclusion

Pregnant patients with symptomatic spinal haemangiomas are a rare phenomenon and require careful, multidisciplinary management. The diagnosis of cord compression can be delayed as a result of multiple clinical distracting factors. A diagnosis of cord compression as a result of a pre-existing haemangioma should be considered in a previously well pregnant patient presenting with lower limb neurological deficits and an apparent reduction in foetal movement. A multidisciplinary approach will certainly improve the outcome.

## Learning points

1. A symptomatic spinal haemangioma in pregnancy is rare.
2. Spinal haemangiomas that present during pregnancy can exhibit typical neurological symptoms of lower limb paraesthesia but can also demonstrate atypical symptoms such as uterine hypoesthesia.
3. MRI is the best imaging modality to diagnose a haemangioma in a pregnant patient.
4. Multidisciplinary team collaboration is an effective tool to improve patient care and outcome.
